# Children’s Eating Behaviour Questionnaire Dimensions and Central Adiposity in Spanish Schoolchildren: Age-Stratified Associations

**DOI:** 10.3390/nu18081283

**Published:** 2026-04-18

**Authors:** Carlos Recio-Añón, Alfonso Lendínez-Jurado, Fernando Mata-Ordóñez, Julia Carracedo-Añón, Antonio González-Martín, María Dolores Marrodán-Serrano

**Affiliations:** 1 Critical Care and Emergency Services Unit, Distrito Sanitario Córdoba Guadalquivir, 14011 Córdoba, Spain; 2 EPINUT Research Group (Ref. 929325), Complutense University of Madrid, 28037 Madrid, Spain; marrodan@ucm.es; 3 UGC Churriana, Distrito Sanitario Málaga-Guadalhorce, 29009 Málaga, Spain; 4 Pediatric Gastroenterology Unit, Vithas Xanit International Hospital, 29630 Málaga, Spain; 5 Instituto de Investigación Biomédica de Málaga (IBIMA)-Plataforma BIONAND, 29010 Málaga, Spain; 6 Department of Pharmacy and Nutrition, Faculty of Biomedical and Health Sciences, Universidad Europea de Madrid, 28670 Madrid, Spain; fernando.mata@universidadeuropea.es; 7 Instituto Maimónides de Investigación Biomédica de Córdoba (IMIBIC), Hospital Universitario Reina Sofía, 14004 Córdoba, Spain; 8 Department of Genetics, Physiology and Microbiology, Faculty of Biological Sciences, Complutense University of Madrid, Health Research Institute Hospital 12 de Octubre (Imas12), 28041 Madrid, Spain; julcar01@ucm.es; 9 Department of Biodiversity, Ecology and Evolution, Faculty of Biological Sciences, Complutense University of Madrid, 28040 Madrid, Spain; antonio@bio.ucm.es

**Keywords:** children’s eating behaviour questionnaire, eating behavior, central adiposity, waist-to-height ratio, KIDMED, pediatrics, schoolchildren

## Abstract

**Background/Objectives:** Elevated central adiposity (ECA) in childhood is associated with early cardiometabolic risk and hemodynamic alterations. However, evidence in Spanish schoolchildren regarding the relationship between eating behavior traits and central adiposity is limited, particularly across developmental stages. This study aimed to examine the association between Children’s Eating Behaviour Questionnaire (CEBQ) subscales and ECA, and to explore potential differences by age group. **Methods:** A cross-sectional study was conducted in 496 rural schoolchildren aged 6–15 years. ECA was defined using the waist-to-height ratio (WHtR) and sex-specific cut-offs validated for the Spanish pediatric population. Eating behavior was assessed with the CEBQ (Z-scores), and diet quality was measured using the KIDMED index. Multivariable logistic regression models were adjusted for sex, KIDMED score, and maternal education. Analyses were subsequently stratified by age (6–9 and 10–15 years). **Results:** The prevalence of ECA was 45.90%. In fully adjusted models, higher Food Responsiveness (FR) was associated with increased odds of ECA, while Satiety Responsiveness (SR) acted as a protective factor; sex also showed an independent association. After stratification, sex remained the only significant predictor in children aged 6–9 years. Among those aged 10–15 years, FR was significantly associated with ECA (*p* = 0.008), while Slowness in Eating (SE) showed a borderline positive association in the adjusted model (*p* = 0.049) and was therefore interpreted cautiously. SR and Emotional Undereating (EU) showed protective trends near significance (*p* = 0.081 and *p* = 0.082, respectively). **Conclusions**: The association between eating behavior traits and ECA varies by age. In older children, FR showed a robust association with ECA, whereas no behavioral predictors were observed in younger children. The protective role of SR in the global model and the emergence of behavioral predictors in older participants highlight the importance of targeted interventions during late childhood.

## 1. Introduction

Childhood obesity is one of the foremost contemporary public health crises. In Spain, the most recent epidemiological data confirm alarmingly high prevalences of excess weight in the pediatric population. Although epidemiological research has historically focused on large urban centers, children living in rural settings and socially vulnerable sectors warrant dedicated attention. Indeed, the recent ALADINO 2023 Study [1] shows that while the overall prevalence of excess weight among 6–9-year-old schoolchildren is 36.10%, a persistent socioeconomic gap remains troubling: obesity among children from lower-income households (23.60%) is roughly double that of those from higher-income homes (10.90%). This gradient in excess weight—linked broadly to socioeconomic barriers such as limited access to fresh foods or a lack of extracurricular offerings—requires specific analysis in decentralized rural areas. It is essential to determine whether, in these settings with distinct food availability and leisure dynamics, such factors operate as specific risks or whether under-studied traditional protective mechanisms exist.

From a metabolic-risk assessment standpoint, body mass index (BMI) has important methodological limitations: it does not distinguish between fat and lean mass, nor does it capture adipose tissue distribution. Central adiposity is a substantially more robust predictor of early hemodynamic alterations and cardiometabolic risk. Tools such as the waist-to-height ratio (WHtR) have demonstrated high sensitivity for detecting such risk in pediatrics [2,3]. Although a universal threshold of 0.50 is often proposed internationally, the use of population-specific references markedly improves diagnostic accuracy. Accordingly, our study employs the sex- and age-specific WHtR cut-offs established by Marrodán et al. for Spanish children and adolescents [4].

Identifying modifiable factors that lead to visceral fat accumulation is critical from a preventive perspective. In this regard, Wardle’s “Behavioral Susceptibility Theory” [5] posits that genetically influenced—but environmentally modifiable—individual differences in appetite are key drivers of weight gain. To quantify these traits, the Children’s Eating Behaviour Questionnaire (CEBQ) has become the reference instrument. Recent studies have validated the high reliability of the CEBQ in Spanish schoolchildren, showing that “food approach” traits (e.g., food responsiveness or emotional overeating) are strongly correlated with obesity [6,7].

Despite the theoretical basis for this relationship, there is a gap in the literature regarding how specific CEBQ dimensions relate to central adiposity in schoolchildren from rural areas. Moreover, eating-behavior phenotypes are not static; the transition from childhood to adolescence involves neurocognitive changes and increasing autonomy that may profoundly modify the relationship between how children eat and the fat they accumulate.

Therefore, the objective of the present study is to evaluate the association between distinct eating-behavior traits (CEBQ) and elevated central adiposity (ECA), defined according to the aforementioned WHtR cut-offs [4] in a rural Spanish pediatric sample, with stratified analyses comparing primary-school age and adolescence.

## 2. Materials and Methods

### 2.1. Study Design and Participants

This cross-sectional observational study was conducted among schoolchildren residing in rural areas of the Campiña de Écija (Seville, Andalusia, Spain). All students enrolled in five educational centers located across three rural municipalities were invited to participate during the 2023–2024 academic year (February–June 2024). The study population comprised children aged 6 to 15 years whose parents or legal guardians provided written informed consent. The study protocol was approved by the Ethics Committee of the Virgen Macarena–Virgen del Rocío University Hospitals (meeting of 21 December 2023; approval record CEI_11/2023 dated 18 January 2024) and adhered to the principles of the Declaration of Helsinki. Of the 585 schoolchildren invited, 497 agreed to participate (response rate: 84.9%). One participant was excluded due to missing CEBQ data, resulting in a final sample of 496 students (249 boys and 247 girls).

### 2.2. Anthropometry and Definition of Central Adiposity

Anthropometric assessments were performed by trained healthcare staff using standardized and calibrated equipment, following the protocols of the International Biological Programme [8] and the International Society for the Advancement of Kinanthropometry [9]. Measurements included weight (kg), height (cm), and waist circumference (cm). Waist circumference was measured in a horizontal plane just above the upper border of the iliac crests, using a non-elastic measuring tape. Based on these measurements, BMI (kg/m^2^) and WHtR were calculated.

Central adiposity was defined according to the sex-specific WHtR cut-offs validated for the Spanish pediatric population by Marrodán et al. [4], which offer greater diagnostic accuracy in this setting compared with the universal 0.50 threshold. Children were classified as having central overweight when WHtR exceeded 0.48 in boys and 0.47 in girls, and central obesity when WHtR exceeded 0.51 in boys and 0.50 in girls. For clinical interpretability and to maximize statistical power in multivariable models, a dichotomous variable—elevated central adiposity—was created by grouping children with central overweight or central obesity versus those with normal WHtR. For global nutritional characterization, BMI categories were also derived using the international cut-offs by Cole et al. [10], ensuring comparability across pediatric populations. Although these Spanish pediatric cut-offs improve diagnostic accuracy in the present setting, direct comparison with studies using the universal WHtR threshold of 0.50 should be made cautiously.

### 2.3. Eating Behavior and Overall Diet Quality

Eating-behavior traits were assessed using the CEBQ [5], completed by parents or legal guardians. This 35-item instrument—previously validated with high reliability in Spanish schoolchildren [6,7]—uses a five-point Likert scale (“never” to “always”). It comprises eight subscales grouped into two major dimensions: food-approach behaviors (Enjoyment of Food, Food Responsiveness, Emotional Overeating, Desire for Drinks) and food-avoidance behaviors (Satiety Responsiveness, Slowness in Eating, Emotional Undereating, Food Fussiness). Reverse-coded items were appropriately recoded.

To minimize data loss while ensuring metric integrity, mean subscale scores were computed. Subscales were retained when at least 80% of items were completed; for subscales meeting this criterion, missing items were imputed using the mean of the completed items within the same subscale, following established CEBQ scoring procedures [11]. After processing, multivariable models included 492 participants, as four did not meet minimum subscale completeness criteria. Raw scores were subsequently converted to Z-scores (mean 0; standard deviation SD-1) to facilitate comparability of effect sizes across subscales.

Overall diet quality was assessed with the KIDMED index, a validated tool to measure adherence to the Mediterranean diet in Spanish children and adolescents [12,13]. This 16-item questionnaire (yes/no responses) was administered via structured face-to-face interviews with participants. Total scores ranged from 0 to 12 and were categorized as high/optimal adherence (≥8), intermediate adherence (4–7), or low/very low adherence (≤3).

### 2.4. Blood Pressure Assessment

Blood pressure was measured in a quiet environment using an oscillometric technique with a pediatric-validated automatic monitor (Microlife WatchBP Office) [14]. Cuff size was selected according to each participant’s arm circumference, ensuring that the inflatable bladder covered 80–100% of the arm’s circumference and at least 40% of its length.

Following European Society of Hypertension recommendations [15], participants rested for 5 min in a seated position with back supported, feet flat on the floor, and the right arm positioned at heart level. Three consecutive measurements were obtained at one-minute intervals, and the mean of the second and third readings was used for analysis. Elevated blood pressure (EBP) was defined as systolic or diastolic pressure ≥ the 90th percentile for age, sex, and height, based on the Spanish RICARDIN II reference tables [16]. Given the cross-sectional design and single-visit protocol, these values represent epidemiological screening estimates rather than confirmatory clinical diagnoses.

### 2.5. Family Variables

Maternal educational level was collected through a self-administered home questionnaire and categorized into three levels: (1) basic education (no schooling or primary education), (2) intermediate education (secondary schooling), and (3) higher education (university studies).

### 2.6. Statistical Analysis

Descriptive characteristics of the sample were presented as mean ± SD for continuous variables and as absolute frequencies and percentages for categorical variables. Descriptions were provided for the total sample and stratified by developmental stage (6–9 years and 10–15 years). Differences in mean Z-scores of the eight CEBQ subscales between children with normal WHtR and those with ECA were examined using independent-samples Student’s *t*-tests. The distribution of CEBQ subscale Z-scores was assessed using histograms, normal Q-Q plots, and the Kolmogorov–Smirnov and Shapiro–Wilk tests, both in the total sample and after age stratification. Although several subscales showed statistically significant deviations from normality, graphical inspection suggested no severe departures, and Student’s *t*-tests were therefore retained for the main bivariate comparisons.

To address the primary objective, binary logistic regression models were constructed with ECA as the dependent variable. The eight CEBQ subscale Z-scores were entered simultaneously as predictors to estimate the independent association of each specific eating-behavior trait with ECA. Accordingly, each coefficient represents the association of a given trait with ECA after adjustment for the remaining CEBQ traits and the selected covariates. The magnitude of associations was expressed as odds ratios (OR) and 95% confidence intervals (95% CI) for each one-SD increase in the predictor. Two models were estimated: an unadjusted model and a multivariable model adjusted simultaneously for sex (reference: girls), Mediterranean diet adherence according to KIDMED (three categories; reference: low adherence), and maternal educational level (three categories; reference: basic education). Given that the relationship between eating behavior and adiposity may differ across maturational stages, analyses were stratified into two age groups (6–9 and 10–15 years) to capture developmental transitions in eating autonomy and avoid masking effects through linear adjustment.

Prior to model construction, severe multicollinearity was ruled out by verifying acceptable tolerance and variance inflation factor (VIF) values. Internal consistency of each CEBQ subscale was evaluated using Cronbach’s alpha. Statistical significance was set at *p* < 0.05. All analyses were performed using IBM SPSS Statistics version 30.0.

## 3. Results

### 3.1. Sample Characteristics

In the total sample analyzed (*n* = 496), the prevalence of ECA was 45.90%, while 5.80% of participants presented elevated blood pressure (≥90th percentile for age, sex, and height). Descriptive characteristics of the study population according to WHtR categories are shown in Table 1. As described in the Section 2, although baseline descriptive analyses were performed on the full sample, multivariable models were conducted with 492 participants due to the exclusion of four subjects who did not meet the minimum completeness criteria required for at least one CEBQ subscale.

### 3.2. CEBQ Subscales by WHtR Categories

**Figure 1:**
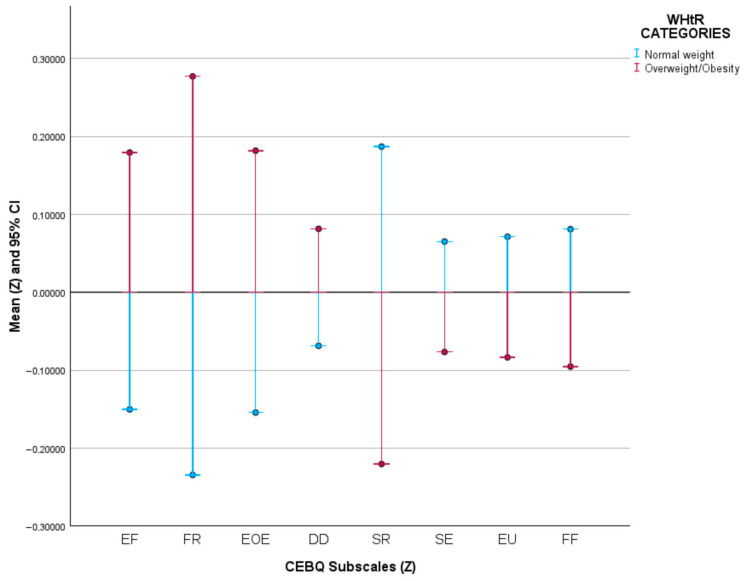
Comparison of CEBQ subscale scores between children with normal and high WHtR. EF: Enjoyment of Food; FR: Food Responsiveness; EOE: Emotional Overeating; DD: Desire to Drink; SR: Satiety Responsiveness; SE: Slowness in Eating; EU: Emotional Undereating; FF: Food Fussiness; WHtR: Waist-to-Height Ratio.

Mean Z-scores of the eight CEBQ dimensions stratified by WHtR categories are presented in Figure 1. Compared with children with normal WHtR, those with ECA showed significantly higher scores in food-approach traits, specifically Enjoyment of Food (EF), Food Responsiveness (FR), and Emotional Overeating (EOE) (all *p* < 0.001). Conversely, Satiety Responsiveness (SR) was significantly lower in the ECA group (*p* < 0.001). Food Fussiness (FF) was slightly lower among children with ECA (*p* = 0.044). No statistically significant differences were observed for Desire to Drink (DD) (*p* = 0.097), Slowness in Eating (SE) (*p* = 0.119), or Emotional Undereating (EU) (*p* = 0.112).

### 3.3. Independent Association Between Eating Behavior and Central Adiposity

Table 2 presents the binary logistic regression analyses conducted on the study sample (*n* = 492). FR was independently associated with higher odds of ECA. This relationship persisted in both crude and adjusted models (sex, diet quality, and maternal education). Each 1-SD increase in FR Z-score was associated with greater odds of ECA. Conversely, SR showed a protective association, indicating that higher sensitivity to internal satiety cues was linked to lower ECA likelihood.

Sex was independently associated with ECA, as detailed in Table 2. No statistically significant independent associations were found for adherence to the Mediterranean diet (KIDMED), maternal education, or the remaining CEBQ subscales when all predictors were entered simultaneously.

Diagnostic analyses revealed no multicollinearity issues (tolerance: 0.39–0.80; VIF: 1.25–2.55). Internal consistency across CEBQ dimensions ranged from Cronbach’s α = 0.64 (EF) to α = 0.89 (FF).

### 3.4. Stratified Analysis by Developmental Stage

Age-stratified models (Table 3) revealed distinct patterns of association between eating behavior and ECA depending on developmental stage.

In adolescents (10–15 years), FR was significantly associated with ECA (*p* = 0.008), and SE also reached nominal statistical significance; however, the association was positive rather than protective (OR = 1.384, 95% CI 1.001–1.912), and should therefore be interpreted cautiously. SR and EU displayed near-significant protective trends (*p* = 0.081 and *p* = 0.082, respectively).

In younger children (6–9 years), none of the CEBQ subscales were significantly associated with ECA. In this group, sex was the only statistically significant predictor. In contrast, sex showed no independent association in the older group.

Collectively, these findings indicate that behavioral components influencing central adiposity become more prominent with age, particularly regarding FR.

## 4. Discussion

Our findings support the validity of the CEBQ in the Spanish context, consistent with evidence of reliability reported in national cohorts, while noting a borderline internal consistency for EF (α = 0.64). Although acceptable for behavioral constructs, this suggests that hedonic enjoyment may contain culture- and setting-specific nuances in rural environments that affect item interpretation. Beyond psychometrics, the present work adds specificity by linking eating-behavior traits to central adiposity rather than BMI alone, thereby addressing body-fat distribution and early cardiometabolic risk more directly.

International literature has validated the CEBQ as a tool to identify obesity risk and relate appetite phenotypes to body composition across pediatric settings, including recent studies in children and adolescents (7–15 years) showing associations between EF or EU and markers of adiposity and fat-free mass [17]. Recent validation evidence from Brazil further supports the applicability of the CEBQ in Latin American pediatric populations. In that study, food-approach traits were positively associated with BMI-for-age, whereas SR showed an inverse association [18]. In US samples, an optimized three-factor structure has been proposed for school-age children with overweight/obesity, underscoring adaptability across anthropometric profiles [19]. In line with calls to go beyond BMI, our use of WHtR offers a more specific diagnostic lens for abdominal fatness and early hemodynamic alterations, cohering with evidence that WHtR can outperform BMI and is comparable or slightly superior to waist circumference in adults [3,20]. In Spanish schoolchildren, WHtR also correlates more strongly than BMI with direct adiposity indicators—percent body fat and skinfold sums—supporting its diagnostic utility [21]. Employing population-specific thresholds [4] strengthens clinical applicability for school health programs. Clinically, the markedly higher prevalence of elevated blood pressure in ECA (9.60% vs. 2.60%) reinforces WHtR as an early-warning screening indicator in pediatrics [22].

Among approach-oriented traits, FR emerged as the predominant correlate of ECA, exceeding EF or EOE. FR reflects a tendency to eat in response to external food cues—such as the sight, smell, availability, or rewarding salience of food—rather than solely internal hunger signals. Within the framework of Behavioral Susceptibility Theory, this finding is consistent with the idea that heightened responsiveness to external food cues may increase susceptibility to excess adiposity in environments where palatable foods are readily available. The attenuation of EF and EOE in the multivariable model may reflect shared variance with FR, suggesting that FR captured the most robust cue-reactive component when all appetitive traits were considered simultaneously. This pattern points to heightened cue reactivity—availability of highly palatable foods, visual prompts, and marketing—as a key driver of central fat accrual in this rural cohort. Such susceptibility frequently bridges toward dysregulated intake, aligning with reviews that identify hedonic processes as early predictors of pediatric obesity risk [23]. A plausible mechanism is eating rate: meta-analytic evidence indicates that fast eating substantially increases the odds of excess weight [24], children with obesity display higher eating speed versus normal-weight peers [25], and “fast eating” has been associated with WHtR ≥ 0.5 in adolescents [26]. It is therefore likely that children with high FR embody a “fast-eater” phenotype, fostering a chronic positive energy balance and preferential abdominal deposition. By contrast, in adolescents aged 10–15 years, SE showed a borderline positive association in the adjusted model (*p* = 0.049), and therefore it should be interpreted cautiously rather than as a clear protective factor. In the same age group, SR and EU showed near-significant protective trends (*p* = 0.081 and *p* = 0.082, respectively), a coherent pattern indicative of better internal regulation under higher environmental demand.

A central contribution of this work is the age-dependent modulation of behavior–adiposity links. In 6–9-year-olds, CEBQ dimensions did not independently predict ECA; sex was the dominant variable (lower odds in boys vs. girls). Early-childhood biology and tighter parental control of the food environment may mask or attenuate the expression of appetite traits, as suggested in preschool research [27]. Still, studies in similarly aged children (6–8 years) report higher EOE under anger or worry among those with obesity [25], indicating that context-specific triggers can already be salient. In contrast, from 10 to 15 years, FR becomes determinative, coinciding with greater autonomy and exposure to out-of-home obesogenic settings. This contrast is compatible with neurodevelopmental maturation and the progressive shift from externally structured to more autonomous eating environments. Emotional eating also tends to emerge in response to pubertal psychosocial stressors—potentially amplified by social media dynamics and internalized body-image norms. As adult supervision wanes, individual appetite phenotypes gain behavioral expression, with more pronounced effects on abdominal fatness [7,25,27].

From a public-health standpoint, identifying FR as a behavioral biomarker enables targeted interventions. Systematic evidence suggests potential to enhance self-regulation via attentive-eating training and meal-context modifications that slow eating [28]; however, in our age-stratified adjusted model, SE was not interpreted as a clear protective factor in adolescents. Nutritional quality remains essential: although our cohort exhibited intermediate adherence to the Mediterranean pattern, recent evidence supports beneficial cardiometabolic effects of high adherence—e.g., on systolic blood pressure and lipids [29]—while other studies highlight marker-specific associations (e.g., overall nutritional status but not uniformly abdominal obesity) [30]. Taken together, these data argue for a dual strategy against ECA in rural schools: (i) improve diet quality and (ii) address cue reactivity and eating tempo (e.g., structured mealtimes, minimized screens, environmental prompts to chew slowly, and portion-awareness).

Strengths include a high participation rate (84.90%), rigorous anthropometry, and internationally comparable definitions of nutritional status [10]. Limitations include the cross-sectional design—precluding causal inference and allowing reverse causality, whereby central adiposity could influence parental reporting—plus potential desirability bias inherent to parent-reported behavior. This limitation may be particularly relevant in older children, whose eating behavior increasingly occurs outside direct parental observation. Nonetheless, caregiver-perceived risk profiles are pivotal for family-centered interventions. Physical activity was not included in final models to avoid over-report bias in minors, prioritizing robustness of CEBQ–ECA associations. This decision is also supported by evidence indicating frequent discrepancies between self-reported and accelerometer-based physical activity in children and adolescents [31]. In addition, although diet quality was assessed using the KIDMED index and maternal education was included as a proxy for socioeconomic position, we did not collect specific information on fast-food consumption, sleep duration, or screen exposure; therefore, residual confounding cannot be excluded. Blood-pressure measures derived from a single visit should be interpreted strictly as screening, not diagnosis. Longitudinal studies are warranted to test whether early modification of FR and eating rate can prevent ECA across the transition to adolescence, and whether combining behavioral modules (attentive eating, pacing) with diet-quality upgrades yields additive benefits on WHtR and hemodynamic markers.

## 5. Conclusions

In this rural school-based cohort, Food Responsiveness was the eating-behavior trait most consistently associated with elevated central adiposity, whereas Satiety Responsiveness showed a protective pattern in the global model. These findings support using WHtR—together with brief assessment of appetite traits—as a pragmatic school-health screening strategy to prioritize targeted, preventive interventions.

## Figures and Tables

**Table 1:** Sociodemographic, anthropometric, and lifestyle characteristics of the total sample and by WHtR categories.

Variable	Total (*n* = 496)	Normal WHtR (*n* = 268)	ECA (*n* = 228)
**Sex**			
Boys	249 (50.20%)	142 (57.02%)	107 (42.98%)
Girls	247 (49.80%)	126 (51.01%)	121 (48.99%)
**Age group**			
6–9 years	248 (50%)	137 (55.24%)	111 (44.76%)
10–15 years	248 (50%)	131 (52.82%)	117 (47.18%)
**Lifestyle and Health**			
Age (years), mean ± SD	9.45 ± 2.12	9.45 ± 2.13	9.42 ± 2.10
BMI (kg/m^2^), mean ± SD	19.06 ± 4.22	16.53 ± 1.96	21.99 ± 4.23
WHtR, mean ± SD	0.49 ± 0.07	0.43 ± 0.02	0.54 ± 0.06
KIDMED (points), mean ± SD	5.91 ± 2.34	6.03 ± 2.39	5.74 ± 2.32
EBP (*n*, %) ≥ p90	29 (5.80%)	7 (2.60%)	22 (9.60%)

Values are mean ± SD or *n* (%). SD: Standard Deviation; BMI: Body Mass Index; WHtR: Waist-to-Height Ratio; *n*: number of participants; EBP: Elevated Blood Pressure.

**Table 2:** Multivariable logistic regression analysis predicting ECA.

Variable	Adjusted OR (95% CI)	*p*
**Sex (boys vs. girls)**	0.667 (0.453–0.980)	0.039
**KIDMED (global)**	—	0.380
- Intermediate vs. Low	0.829 (0.479–1.434)	0.503
- High vs. Low	0.653 (0.349–1.223)	0.183
**Maternal Education (global)**	—	0.539
- Intermediate vs. Low	1.000 (0.619–1.615)	0.999
- High vs. Low	0.779 (0.448–1.355)	0.377
**Eating Behavior**		
- Enjoyment of Food (EF)	1.152 (0.863–1.539)	0.337
- Food Responsiveness (FR)	1.494 (1.097–2.034)	0.011
- Emotional Overeating (EOE)	1.118 (0.834–1.498)	0.457
- Desire to Drink (DD)	0.985 (0.796–1.217)	0.886
- Satiety Responsiveness (SR)	0.751 (0.564–0.999)	0.049
- Slowness in Eating (SE)	1.146 (0.924–1.421)	0.215
- Emotional Undereating (EU)	0.856 (0.684–1.071)	0.174
- Food Fussiness (FF)	1.095 (0.854–1.403)	0.476

Models adjusted for sex (reference: girls), KIDMED adherence (reference: low), and maternal education (reference: basic).

**Table 3:** Multivariable logistic regression analysis predicting ECA, stratified by age group. Models adjusted for sex (reference: girls), KIDMED adherence (reference: low), and maternal education (reference: basic).

Variable	6–9 Years OR (95% CI)	*p*	10–15 Years OR (95% CI)	*p*
**Sex (boys vs. girls)**	0.469 (0.273–0.806)	0.006	0.885 (0.495–1.581)	0.680
**KIDMED (global)**	—	0.864	—	0.174
- Intermediate vs. Low	0.902 (0.444–1.833)	0.776	0.544 (0.211–1.404)	0.208
- High vs. Low	0.787 (0.329–1.886)	0.591	0.380 (0.135–1.067)	0.066
**Maternal Education (global)**	—	0.609	—	0.737
- Intermediate vs. Low	0.812 (0.406–1.624)	0.556	1.234 (0.607–2.510)	0.561
- High vs. Low	0.671 (0.305–1.474)	0.320	0.972 (0.426–2.219)	0.947
**Eating Behavior**				
- Enjoyment of Food (EF)	1.287 (0.859–1.929)	0.221	1.066 (0.684–1.662)	0.777
- Food Responsiveness (FR)	1.397 (0.938–2.079)	0.100	2.091 (1.217–3.591)	0.008
- Emotional Overeating (EOE)	0.930 (0.608–1.425)	0.740	1.223 (0.786–1.903)	0.373
- Desire to Drink (DD)	1.127 (0.839–1.514)	0.427	0.831 (0.591–1.167)	0.285
- Satiety Responsiveness (SR)	0.869 (0.581–1.301)	0.495	0.678 (0.439–1.049)	0.081
- Slowness in Eating (SE)	0.987 (0.724–1.345)	0.934	1.384 (1.001–1.912)	0.049
- Emotional Undereating (EU)	0.980 (0.709–1.355)	0.903	0.745 (0.535–1.038)	0.082
- Food Fussiness (FF)	1.195 (0.850–1.682)	0.305	1.034 (0.703–1.522)	0.864

## Data Availability

The original data presented in the study are included in the article; further inquiries can be directed to the corresponding authors.

## Abbreviations

The following abbreviations are used in this manuscript:

BMI	Body mass index
WHtR	Waist-to-height ratio
CEBQ	Children’s Eating Behavior Questionnaire
ECA	Elevated central adiposity
SD	Standard deviation
EBP	Elevated blood pressure
OR	Odds ratios
CI	Confidence intervals
VIF	Variance inflation factor
EF	Enjoyment of food
FR	Food responsiveness
EOE	Emotional overeating
SR	Satiety responsiveness
FF	Food fussiness
DD	Desire to drink
SE	Slowness in eating
EU	Emotional undereating
